# 1-Aminocyclopropane-1-Carboxylate and Its Chemoreceptor Drive Metabolic Reprogramming to Enhance Chemotactic Rhizocompetence in *Pseudomonas* sp. UW4

**DOI:** 10.3390/microorganisms14081632

**Published:** 2026-07-27

**Authors:** Tao Li, Bingke Sun, Wei Zhong, Ran Chai, Yingbin Li, Haopeng Chai, Yanan Li, Liyou Qiu, Yuqian Gao

**Affiliations:** 1 College of Applied Engineering, Henan University of Science and Technology, Sanmenxia 472000, China; litao83929@163.com (T.L.); zhongweiwm@163.com (W.Z.); 2 College of Food Landscape Architecture, Sanmenxia Polytechnic, Sanmenxia 472000, China; 3 College of Life Sciences, Henan Agricultural University, Zhengzhou 450046, China; 18303701929@163.com (B.S.); liyingbin2001@163.com (Y.L.); 17637936592@163.com (H.C.); liyanan@henau.edu.cn (Y.L.); 4 Key Laboratory of Enzyme Engineering of Agricultural Microbiology, Ministry of Agriculture and Rural Affairs, Zhengzhou 450046, China; 5 Henan Engineering Technology Research Center of Green Coating Materials, Yellow River Conservancy Technical University, Kaifeng 475004, China; chairan@yrctu.edu.cn

**Keywords:** 1-aminocyclopropane-1-carboxylate, chemoreceptor, chemotaxis, PGPR, proteomics, rhizosphere colonization

## Abstract

1-Aminocyclopropane-1-carboxylate (ACC) has been identified as the preferred chemoattractant driving rhizosphere colonization by the model plant growth-promoting rhizobacterium (PGPR) *Pseudomonas* sp. UW4. However, the regulatory mechanisms governing the expression of its ACC chemoreceptor WP116, as well as how changes in its abundance influence ACC chemotaxis and rhizosphere colonization, remain poorly understood. In this study, we found that the addition of ACC (3.0 mM) during the mid-logarithmic growth phase of UW4 rapidly induced the transcription of *WP116* (up to 1.4-fold) and several other chemoreceptor genes, promoted bacterial growth by increasing the maximum specific growth rate by 21.1% (*p* < 0.05), yet did not significantly alter the protein abundance of chemoreceptors. At the proteome level, however, ACC drove a reprogramming of carbon, nitrogen, and energy metabolism, facilitating the transition of cells from a motile to a colonizing state. Furthermore, trans-overexpression of *WP116* disrupted the homeostatic balance of receptor abundance, leading to a marked increase in its protein level (up to 32.3-fold) and enhancing both the ACC chemotactic response (up to 3–5-fold) and the rhizosphere colonization competitiveness (up to 9-fold) of UW4 (*p* < 0.05). These findings not only deepen our understanding of the molecular mechanisms underlying metabolism-dependent chemotaxis but also provide a novel technological avenue for rhizosphere microbiome engineering aimed at directionally enhancing the chemotactic rhizocompetence of PGPR.

## 1. Introduction

Bacterial chemotaxis is a fundamental sensory behavior that enables cells to detect and respond to chemical gradients, guiding them toward favorable environments or away from harmful substances [1]. This process is mediated by a highly conserved signal transduction system in which transmembrane chemoreceptors, known as methyl-accepting chemotaxis proteins (MCPs), bind extracellular ligands and transmit signals to the CheA–CheW histidine kinase complex. These receptors assemble into large supramolecular arrays at the cell poles, a structural organization that provides the system with remarkable signal amplification, cooperativity, and high sensitivity [2,3,4]. While the receptor structures and core signaling mechanisms have been extensively elucidated, the extent to which variations in chemoreceptor abundance modulate the chemotactic response remains to be systematically investigated.

The expression levels of different types of MCPs vary significantly across various bacterial species. In *Escherichia coli*, for example, the levels of the high-abundance receptors Tar and Tsr are 10- to 30-fold higher than those of the low-abundance receptors Trg and Tap [5,6]. In *Sinorhizobium meliloti*, the ratio of high-, low-, and extremely low-abundance receptors is approximately 300:30:1 [7]. Furthermore, the quantification of ten chemoreceptors in *Bacillus subtilis* revealed a difference in abundance of approximately 40-fold [8]. Similarly, the discrepancy between the highest and lowest expression levels among the chemoreceptors of *Pseudomonas putida* KT2440 reaches 174-fold [9].

The abundance of bacterial chemoreceptors is influenced by medium composition and growth phase. The corresponding chemoeffectors exert three distinct effects on the expression of chemoreceptor genes in *Pseudomonas putida* KT2440. First, chemoeffectors that serve as carbon and/or nitrogen sources induce gene expression. Second, chemoeffectors that bind to two inorganic phosphate-sensing receptors lead to significantly reduced expression. Third, chemoeffectors that bind to three receptors of TCA cycle intermediates do not affect their expression. Notably, a large number of receptors are induced in a minimal growth medium and during the stationary phase [9]. Furthermore, under low concentrations of maize root exudates, the transcript levels of ten out of twenty-seven chemoreceptor genes in *P. putida* KT2440 are upregulated. Conversely, at high concentrations of root exudates, the transcript levels of nearly all receptor genes decrease [10]. In *P. aeruginosa* PAO1, the abundance of three chemoreceptors (PctC, McpK, and PctD) is modulated by phosphate concentration, a non-chemoeffector in the medium [11].

Numerous studies demonstrate that while the affinity between a chemoeffector and its cognate chemoreceptor determines the onset of response, the intracellular abundance of the receptor appears to govern chemotactic magnitude, as evidenced by the enhancement of chemotaxis upon receptor overexpression [12,13,14,15]. Nevertheless, conflicting evidence exists; for example, the abundances of three chemoreceptors (PctC, McpK, and PctD) in *P. aeruginosa* PAO1 showed no correlation with the chemotactic intensity toward their respective ligands [11]. Furthermore, in complex environments where multiple chemoeffectors coexist, the resulting chemotactic output is often dictated by the stoichiometric ratio of the corresponding receptors rather than their absolute concentrations [16,17].

*Pseudomonas* sp. UW4 is a model plant growth-promoting rhizobacterium (PGPR) characterized by its ability to produce 1-aminocyclopropane-1-carboxylate (ACC) deaminase (AcdS) and utilize ACC as a sole carbon and nitrogen source [18,19,20]. Notably, ACC serves as the preferred chemoattractant for UW4, triggering a metabolism-dependent chemotactic response mediated by the MCP WP116. This chemotactic response plays a pivotal role in rhizosphere colonization and consequent plant growth-promoting efficacy [21]. However, several key questions remain to be elucidated: whether ACC regulates *WP116* expression at the transcriptional and translational levels, thereby determining receptor abundance, and whether elevated WP116 protein levels are sufficient to enhance ACC-directed chemotactic responses. The present study was conducted to address these gaps.

## 2. Materials and Methods

### 2.1. Strains and Plasmids

*Pseudomonas* sp. UW4 was kindly provided by Professor Bernard R. Glick from the University of Waterloo, Canada. pRK2013, pBBR1MCS-2, and pBBR1MCS2-pAmp-EGFP [22] were purchased from Shhebio, Shanghai, China.

### 2.2. Growth Curve

Bacteria were cultured in 250 mL Erlenmeyer flasks containing 100 mL of LB medium at 30 °C with shaking at 220 rpm. Optical density (OD_600 nm_) was measured at 2 h intervals. To evaluate the impact of ACC on bacterial growth and chemoreceptor expression, the culture was grown to an OD_600 nm_ of approximately 0.6 and then divided into two equal portions. One portion served as the untreated control, while the other was supplemented with 3.0 mM ACC before further incubation.

### 2.3. Quantitative Real-Time PCR (RT-qPCR)

Bacterial cells were harvested from shake-flask cultures, and total RNA was extracted using RNAiso Plus (Takara Bio, Beijing, China) according to the manufacturer’s instructions. cDNA was synthesized using the HiScript III RT SuperMix for qPCR (+gDNA wiper) (Vazyme, Nanjing, China), which included a genomic DNA removal step. The expression levels of 15 annotated chemoreceptors containing extracellular ligand-binding domains (LBDs) in the *Pseudomonas* sp. UW4 genome [20,21] were analyzed. The *gyrB* gene served as the internal housekeeping control, and all primers used are listed in Table S1. qPCR assays were performed using ChamQ Universal SYBR qPCR Master Mix (Vazyme, Nanjing, China) with the following cycling conditions: initial denaturation at 94 °C for 30 s, followed by 30 cycles of 95 °C for 10 s and 60 °C for 30 s. Relative gene expression levels were calculated using the $2^{-\Delta \Delta C_{T}}$ method.

### 2.4. Proteomics

Bacterial total protein extraction and peptide preparation were performed using the Majorbio microprotein kit (Majorbio, Shanghai, China). Briefly, the samples were suspended in Extract Reagent (Reagent 1) and homogenized via non-contact sonication at a low temperature for 10 min. After high-speed centrifugation (16,000 g, 10 min), the supernatant containing the protein solution was collected. For reduction and alkylation, Diluent (Reagent 2) and Reagent 3 were added to the supernatant, and the mixture was incubated at 55 °C for 30 min. Subsequently, Alkylate Reagent (Reagent 4) was added, and the alkylation reaction proceeded in the dark at room temperature for 30 min. The samples were then digested by adding Digest Reagent (Reagent 5) at 37 °C for 16 h. Finally, Acidize Reagent (Reagent 6) was added for acidification at 45 °C for 30 min to obtain the peptide solution. The peptides were desalted using C18 Tips, and the eluates were dried in a vacuum concentrator. The dried peptides were reconstituted in 0.1% formic acid and quantified by UV spectrophotometry using a NanoDrop One instrument (Thermo Fisher Scientific, Waltham, MA, USA).

LC–MS/MS analysis was conducted on a Vanquish Neo UHPLC system (Thermo Fisher Scientific, Waltham, MA, USA) equipped with a uPAC High Throughput column (75 μm × 5.5 cm, Thermo Fisher Scientific, Waltham, MA, USA). Mobile phase A consisted of 0.1% formic acid and 2% acetonitrile in water, and mobile phase B consisted of 0.1% formic acid and 80% acetonitrile in water. Mass spectrometry was performed on a timsTOF Ultra 2 mass spectrometer (Bruker, Bremen, Germany) operating in Data-Independent Acquisition (DIA) mode. The instrument was operated in positive ion mode with an ion source voltage of 4.5 kV and a mass scan range of 100–1700 *m*/*z*.

Proteins were identified using Spectronaut™ 19, with the identification criteria set to a Protein/Peptide false discovery rate (FDR) ≤ 0.01, an extracted ion chromatogram (XIC) width ≤ 75 ppm, and requiring ≥ 1 unique peptide. Protein quantification was performed using the MaxLFQ algorithm. Relative quantification was performed using MS1 signal intensity for label-free quantification (LFQ). Differential expression thresholds were defined as a fold change (FC) > 1.5 or <0.67 and *p* < 0.05 (Student’s *t*-test). Functional enrichment analysis (GO/KEGG) and protein–protein interaction (PPI) networks (STRING v11.5) were conducted via the Majorbio Cloud platform.

### 2.5. Construction of Bacterial Strain

The construction of the *Pseudomonas* sp. UW4 strain for the fusion expression of the ACC chemoreceptor WP116 and EGFP was carried out using derivative plasmids of pBBR1MCS2. Three fragments, namely wp116, the (G4S)3 linker, and EGFP, with homology arms, were PCR-amplified using the UW4 genome, pBBR1MCS2-pAmp-EGFP, and a synthetic (G4S)3 linker as templates, respectively. The primers used are listed in Table S2. These three fragments were then digested and ligated to obtain a fusion fragment, which was cloned into the plasmid pBBR1MCS2 to generate the recombinant plasmid pBBR1MCS2-WP116-EGFP. The resulting plasmid was introduced into UW4 by triparental mating [18,23] to obtain the strain UW4/pOE-*WP116*-*EGFP*.

### 2.6. Subcellular Localization

Live bacterial cells were immobilized for imaging using a modified agarose pad method [24]. Briefly, agarose pads were prepared by solidifying LB medium containing 1.5% agarose on microscope slides. A bacterial suspension was applied to the pad surface and covered with a coverslip. Fluorescence images of WP116-EGFP were acquired using a confocal laser scanning microscope (Nikon A1HD25, Nikon, Tokyo, Japan) with excitation at 488 nm and emission collected at 509 nm.

### 2.7. Chemotaxis Assays

Chemotaxis drop assay was performed as previously described [25] with minor modifications. Briefly, exponentially growing bacterial cells were harvested, washed, and resuspended in chemotaxis buffer. Hydroxypropyl methylcellulose was added to the suspension to a final concentration of 0.2% (*w*/*v*), and 10 mL of the mixture was poured into a Petri dish (90 mm). Subsequently, 10 μL of 0.5 M ACC was applied to the center of the plate. The plates were left undisturbed at room temperature for 2–4 h, and the diameter of the resulting chemotactic ring was measured.

High-throughput capillary chemotaxis assay was performed using a previously described method [21,26], with minor modifications. Briefly, 300 μL of bacterial suspension (OD_600 nm_ = 0.2) prepared in chemotaxis buffer was dispensed into each well of a sterile 96-well microplate. Separately, 100 μL of chemotaxis buffer or ACC solutions (10^−3^, 10^−4^, or 10^−5^ M) was added to the wells of another 96-well microplate, sterile 1-μL capillaries were filled to approximately one-third of their length, and the opposite ends were sealed with capillary sealing wax. The sealed ends of the capillaries were inserted into the wells of a 96-well plate containing 3% (*w*/*v*) solidified agar to secure their positions. The plate assembly was then inverted so that the open ends of the capillaries were immersed in the corresponding wells containing the bacterial suspensions. Chemotaxis was allowed to proceed at room temperature for 30 min, and bacteria from each capillary were quantified by the plate-counting method to determine the number of CFU.

### 2.8. Competitive Root Colonization Assays

Wheat seeds were sterilized and germinated as described previously [27]. Germinated seeds were then placed at the center of 50 mL sterile tubes containing 40 g of sterile washed silica sand. For competitive colonization assays, a mixed suspension of UW4/pOE-*WP116*-*EGFP* and the empty-vector control strain was inoculated at the edge of each tube as described previously [14]. After 10 days of cultivation, the bacterial populations in the rhizosphere or within 1 mm of the root apex were determined using a plate dilution method on LB-agar supplemented with 100 μg/mL of ampicillin and kanamycin. Total colonies represented the combined population of UW4/pOE-*WP116*-*EGFP* and the empty-vector control strain. Fluorescent colonies were counted under blue-light excitation as UW4/pOE-*WP116*-*EGFP*. The number of empty-vector control colonies was calculated by subtracting the fluorescent colonies from the total.

### 2.9. Data Analysis

Data processing and curve fitting were carried out using GraphPad Prism 10.2.2. Student’s *t*-test was used for paired comparisons between two different groups. Bacterial growth curve was modeled with the Logistic equation

Y = YM × Y0/((YM − Y0) × exp(−k × x) + Y0)

where Y0 represents the value at zero, YM the maximum population, and k the rate constant (maximum specific growth rate, *μ*_max_).

## 3. Results

### 3.1. Impact of ACC on Growth and Chemoreceptor Gene Expression in Pseudomonas sp. UW4

**Figure 1:**
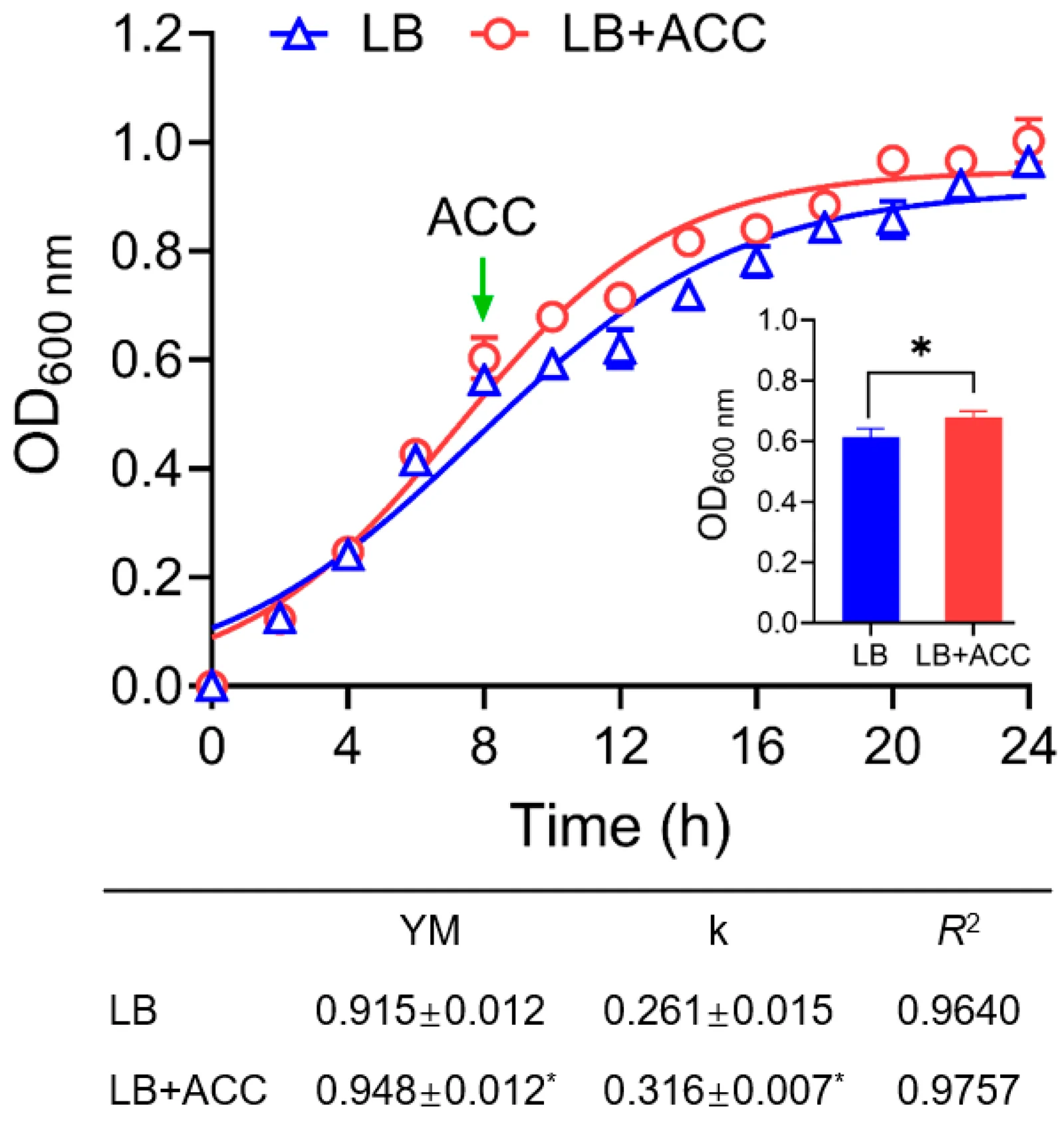
ACC promotes the flask-culture growth of *Pseudomonas* sp. UW4. The main graph shows the Logistic-fitted growth curves of UW4 cultured in LB medium and LB supplemented with ACC (LB + ACC). The arrow marks the addition of ACC. The inset shows the comparison of OD values at 45 min after ACC addition. LB, Lysogeny Broth medium; ACC, 1-aminocyclopropane-1-carboxylate. The table below summarizes the growth kinetic parameters fitted by the Logistic equation: YM represents the maximum biomass; k represents the maximum specific growth rate, and *R*^2^ is the coefficient of determination. Data are expressed as mean ± SD (n = 3). * *p* < 0.05 determined by Student’s *t*-test on paired samples.

**Figure 2:**
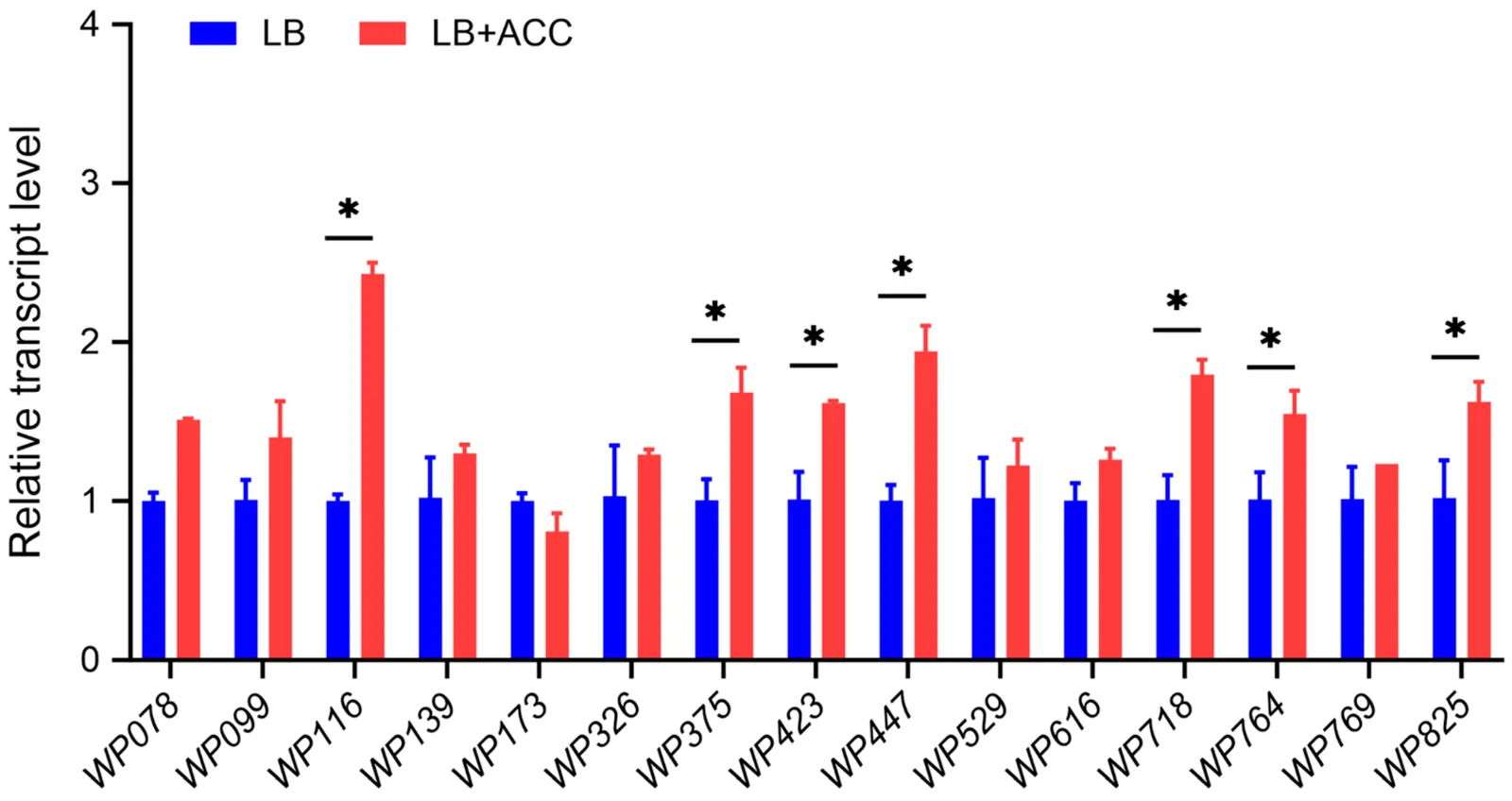
ACC regulated the expression of extracellular LBD-containing chemoreceptor genes in *Pseudomonas* sp. UW4. The NCBI accession numbers of the genes are provided in Table S1. Relative gene expression levels measured by quantitative PCR at 45 min after ACC addition during the logarithmic growth phase. LB, Lysogeny Broth medium; ACC, 1-aminocyclopropane-1-carboxylate. Data are expressed as mean ± SD (n = 3). * *p* < 0.05 determined by Student’s *t*-test on paired samples.

To investigate the effects of ACC on bacterial growth and the expression of chemoreceptor genes, *Pseudomonas* sp. UW4 was cultured in shake flasks to the mid-logarithmic growth phase, followed by the addition of 3.0 mM ACC. After 45 min of treatment, the OD_600 nm_ of the culture increased by 11% compared to the untreated control. Subsequent monitoring revealed an altered growth curve (Figure 1), with the ACC treatment exhibiting a 21.1% increase in the maximum specific growth rate (*μ*_max_) and a 3.6% increase in the maximum biomass. Furthermore, cells harvested at 45 min post-treatment were analyzed for the expression of chemoreceptor genes containing extracellular LBDs. Compared to the control, seven chemoreceptor genes were upregulated, among which the specific ACC chemoreceptor gene, *WP116*, showed the highest upregulation, reaching a 1.4-fold increase (Figure 2). Furthermore, *gyrB* was stably expressed under ACC treatment and was therefore suitable for RT-qPCR normalization (Figure S1).

### 3.2. Proteomic Analysis of Differentially Expressed Proteins in Pseudomonas sp. UW4 upon ACC Treatment

Proteomic analysis was performed on *Pseudomonas* sp. UW4 samples collected 45 min post-ACC addition during the mid-logarithmic growth phase. A total of 3859 proteins were identified across the ACC-treated and untreated control groups (Table S3). A total of 41 differentially expressed proteins (DEPs) were identified, including 34 upregulated and 7 downregulated proteins (Table 1).

Compared to the untreated control, the expression levels of all chemoreceptors and chemotaxis-related proteins showed no significant changes upon ACC treatment. The significantly upregulated proteins were primarily involved in the following biological processes: (1) Carbon and ACC metabolism, including the key pentose phosphate pathway (PPP) enzyme transketolase and the key ACC metabolism enzyme ACC deaminase; (2) The TCA cycle and oxidative metabolism, such as dihydrolipoyllysine-residue acetyltransferase, alpha-ketoglutarate dehydrogenase, and formate dehydrogenase-N subunit alpha; (3) Aromatic amino acid/compound degradation (homogentisate pathway), including homogentisate 1,2-dioxygenase, fumarylacetoacetase, 4-hydroxyphenylpyruvate dioxygenase, and maleylacetoacetate isomerase; (4) Colonization, attachment, and biofilm formation, such as the Flp pilus assembly protein CpaB and retention module-containing protein; and (5) Acute protein folding and oxidative stress responses, including thioredoxin family protein, peptidylprolyl isomerase, and TolC.

Conversely, the significantly downregulated proteins were mainly associated with flagellar assembly and motility (flagellar assembly peptidoglycan hydrolase FlgJ), cell wall synthesis and cell division (FtsW and MurJ), and cell membrane and surface antigen synthesis (YidD and O-antigen ligase family protein).

### 3.3. Overexpression of the ACC Chemoreceptor WP116 Enhances Protein Abundance

**Figure 3:**
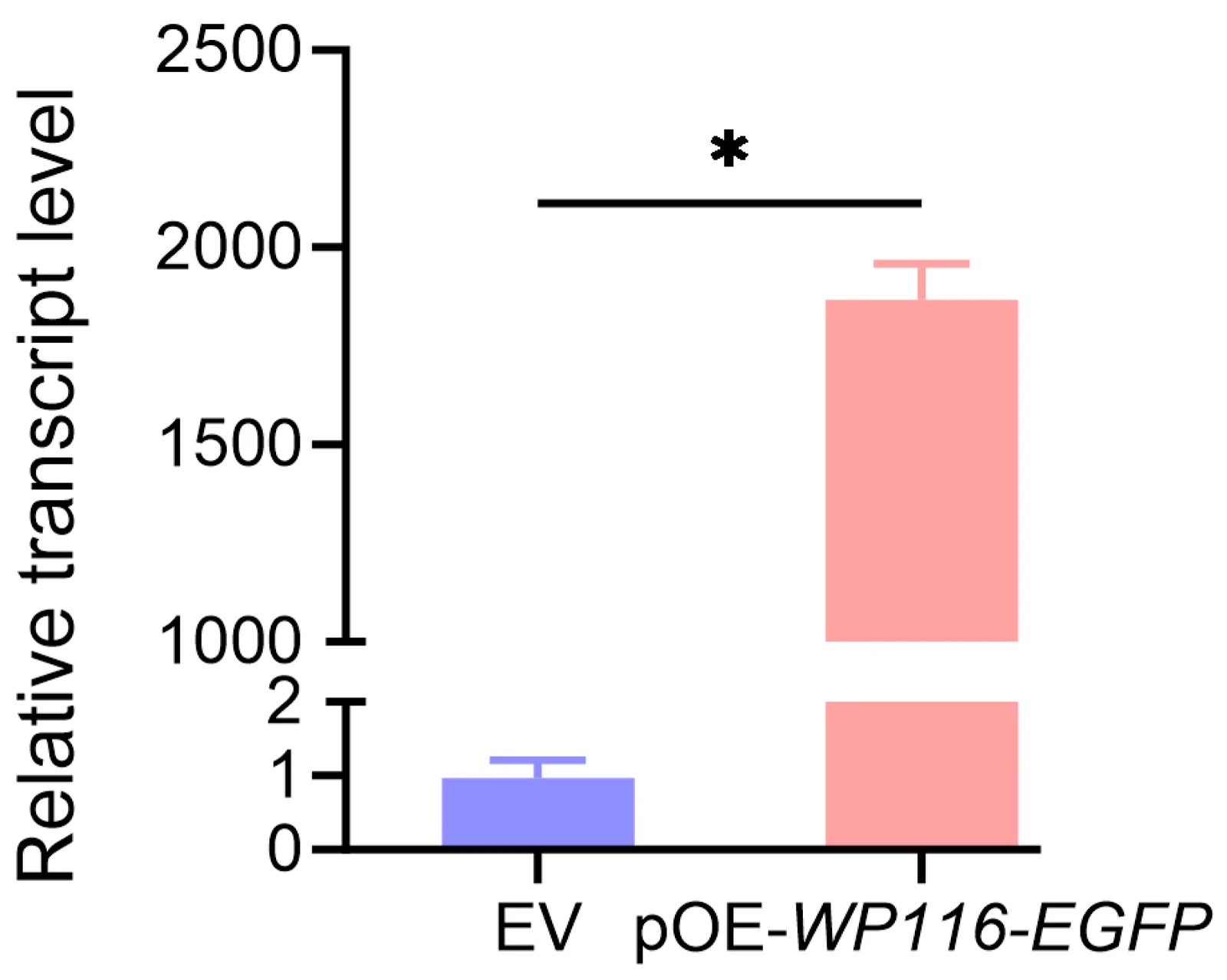
Relative transcript levels of ACC chemoreceptor *WP116* in *Pseudomonas* sp. UW4 strains. EV, empty-vector control strain; pOE-*WP116*-*EGFP*, *WP116*-overexpression strain. Data are expressed as mean ± SD (n = 3). * *p* < 0.05 determined by Student’s *t*-test on paired samples.

**Figure 4:**
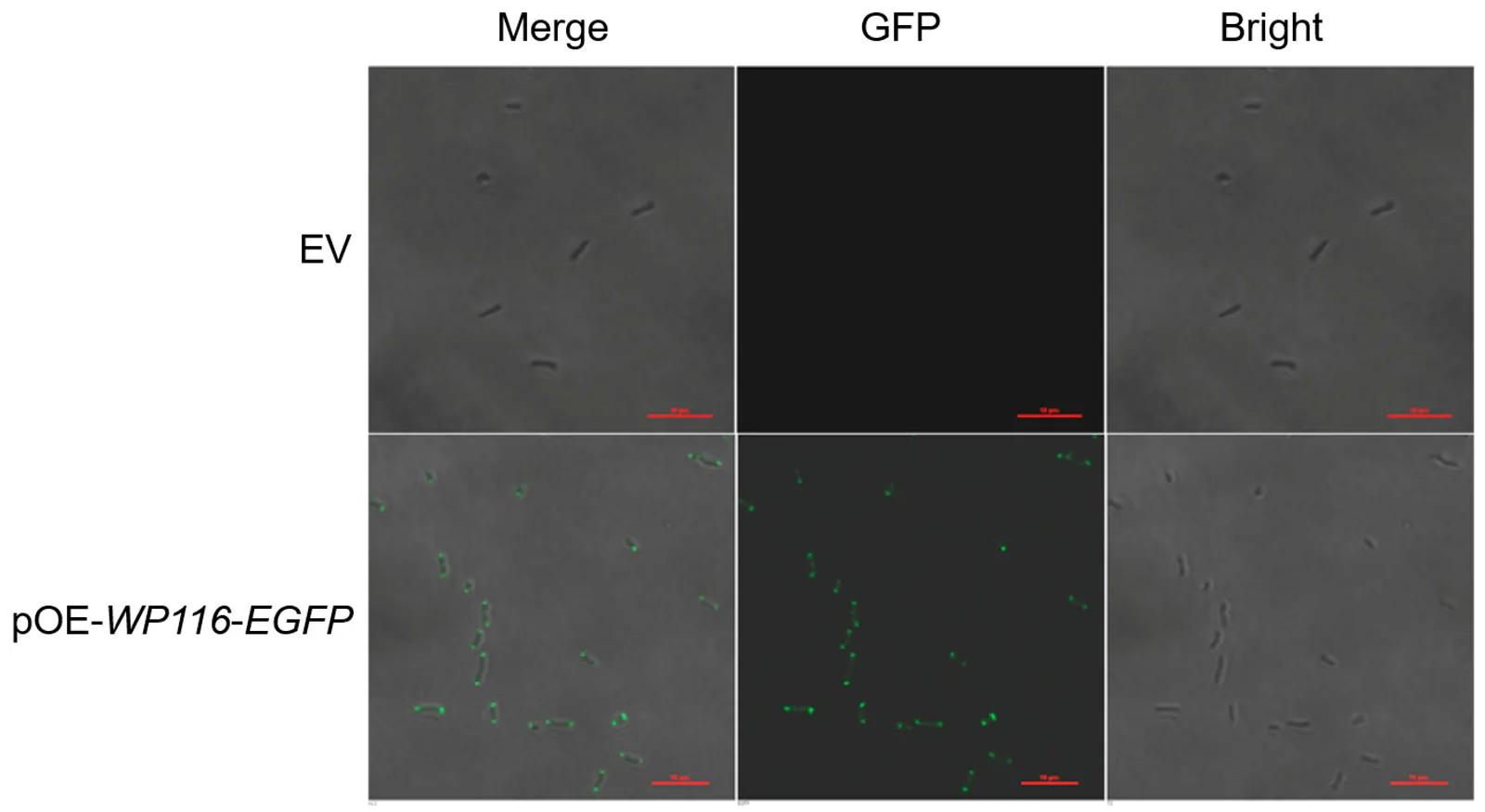
The subcellular localization of ACC chemoreceptor WP116 in *Pseudomonas* sp. UW4 strains. EV, empty-vector control strain; pOE-*WP116*-*EGFP*, *WP116*-overexpression strain. GFP, Bright, and Merge channels were labeled on the top of the pictures. Scale bars, 10 μm.

The *Pseudomonas* sp. UW4 strain overexpressing the ACC chemoreceptor *WP116* (UW4/pOE-*WP116*-*EGFP*) and its corresponding empty-vector control were cultured in LB medium in shake flasks to mid-exponential phase (OD_600 nm_ = 0.6). Quantitative PCR (qPCR) analysis confirmed that *WP116* transcript levels in the overexpression strain were approximately 2000-fold higher than in the control (Figure 3). Confocal laser scanning microscopy revealed that the WP116-EGFP fusion protein predominantly localized at the cell poles, aligning with the typical distribution of chemoreceptors; no detectable fluorescence was observed in the control (Figure 4).

**Figure 5:**
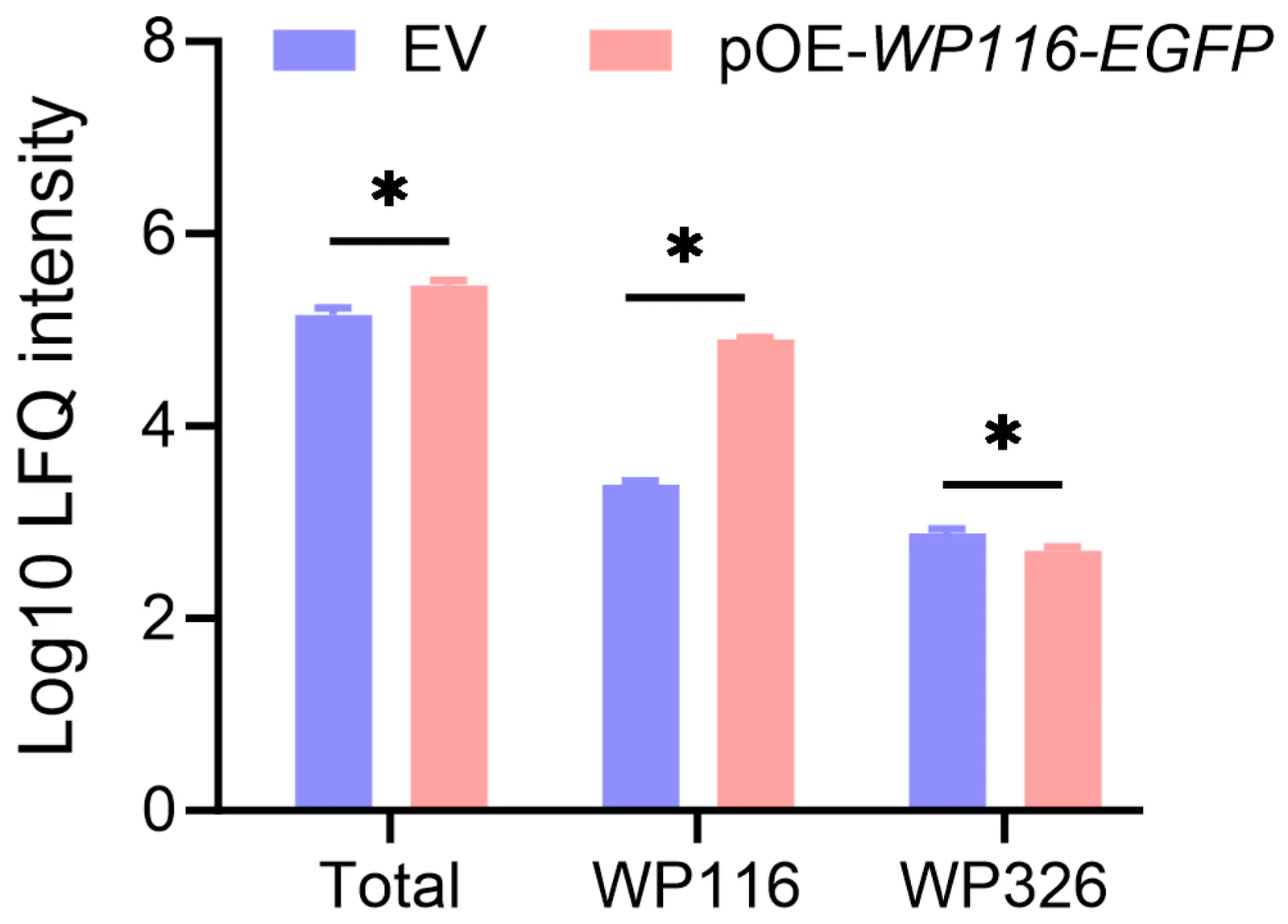
Abundance of chemotaxis receptors in *Pseudomonas* sp. UW4 strains. EV, empty-vector control strain; pOE-*WP116*-*EGFP*, *WP116*-overexpression strain; Total, sum of the abundance of all detected chemoreceptors; WP326, chemotaxis receptor. Data are expressed as mean ± SD (n = 3). * *p* < 0.05 determined by Student’s *t*-test on paired samples.

Proteomic analysis was performed on samples collected during the mid-logarithmic growth phase (OD_600 nm_ = 0.6) to compare the *Pseudomonas* sp. UW4 *WP116*-overexpression strain with the empty-vector control strain. A total of 3953 proteins were identified across both strains (Table S4). Proteomic profiling of chemoreceptor abundance in UW4 strains revealed that, compared to the empty-vector control, only WP116 was significantly upregulated in the *WP116*-overexpression strain, reaching a level 32.3-fold higher than that of the control. Among the remaining receptors, only WP326 exhibited a significant decrease of 34.4%. Consequently, the total abundance of all chemoreceptors in the overexpression strain was 2.0 times that of the control strain, with the proportion of WP116 receptor within the total receptor pool increasing from 1.7% to 27.3% (Figure 5).

### 3.4. Overexpression of the ACC Chemoreceptor WP116 Enhances Chemotaxis Toward ACC and Competitive Rhizosphere Colonization in Pseudomonas sp. UW4

**Figure 6:**
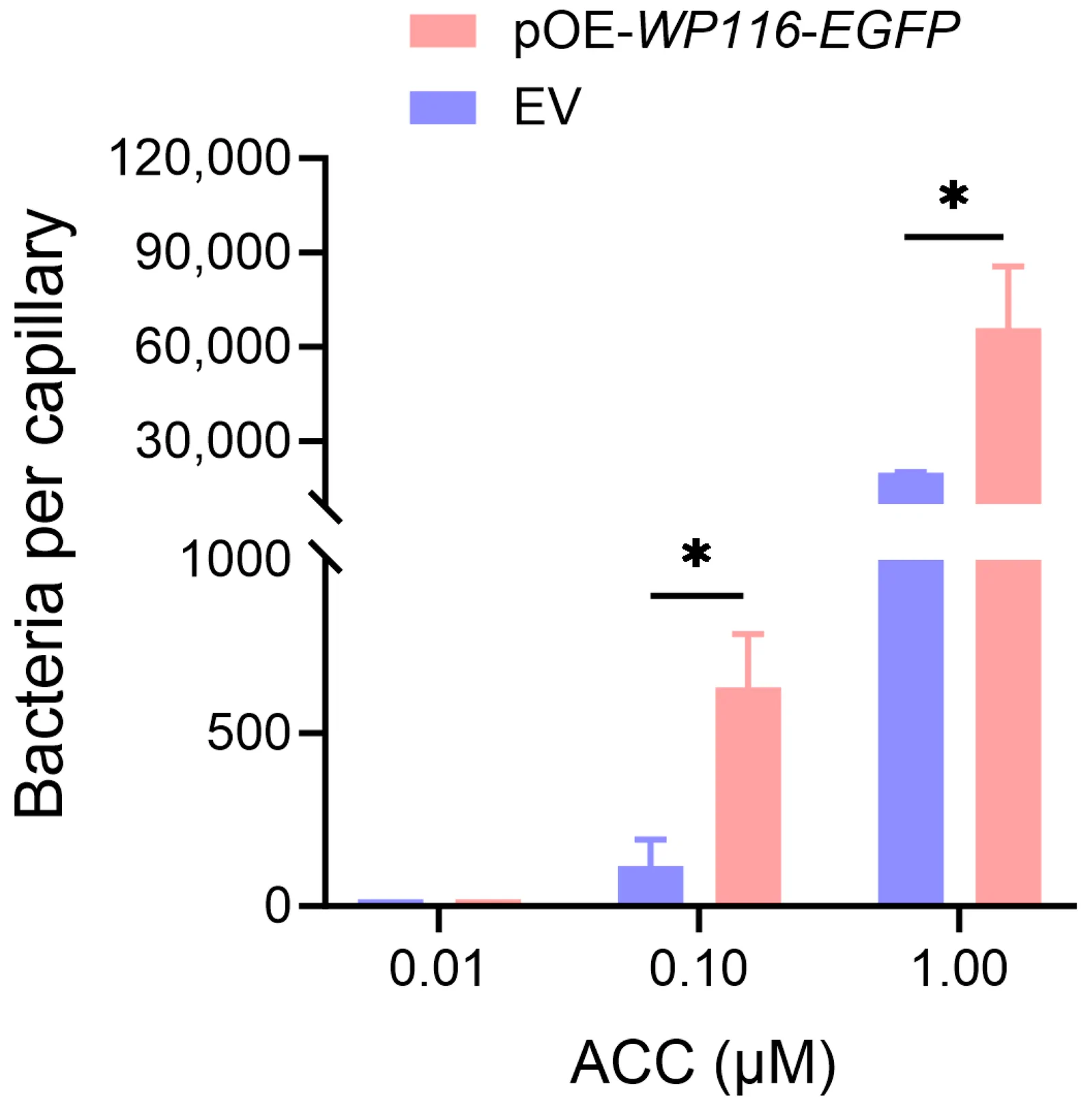
Quantitative capillary chemotaxis assays of *Pseudomonas* sp. UW4 strains toward ACC. ACC, 1-aminocyclopropane-1-carboxylate; EV, empty-vector control strain; pOE-*WP116*-*EGFP*, *WP116*-overexpression strain. Data were normalized by subtracting the number of cells that migrated into buffer-filled capillaries and are presented as mean ± SD (n = 3). * *p* < 0.05 determined by Student’s *t*-test on paired samples.

To evaluate whether WP116 overexpression enhances ACC chemotaxis in *Pseudomonas* sp. UW4, quantitative capillary chemotaxis assays were performed. Both the empty-vector control and *WP116*-overexpression strains were highly sensitive to ACC, showing detectable chemotaxis at concentrations as low as 0.1 μM. At 1.0 μM ACC, the chemotactic response increased by more than 100-fold in both strains. Notably, compared with the empty-vector control, the *WP116*-overexpression strain displayed a more than 5-fold stronger chemotactic response at 0.1 μM ACC and a more than 3-fold stronger response at 1.0 μM ACC (Figure 6).

**Figure 7:**
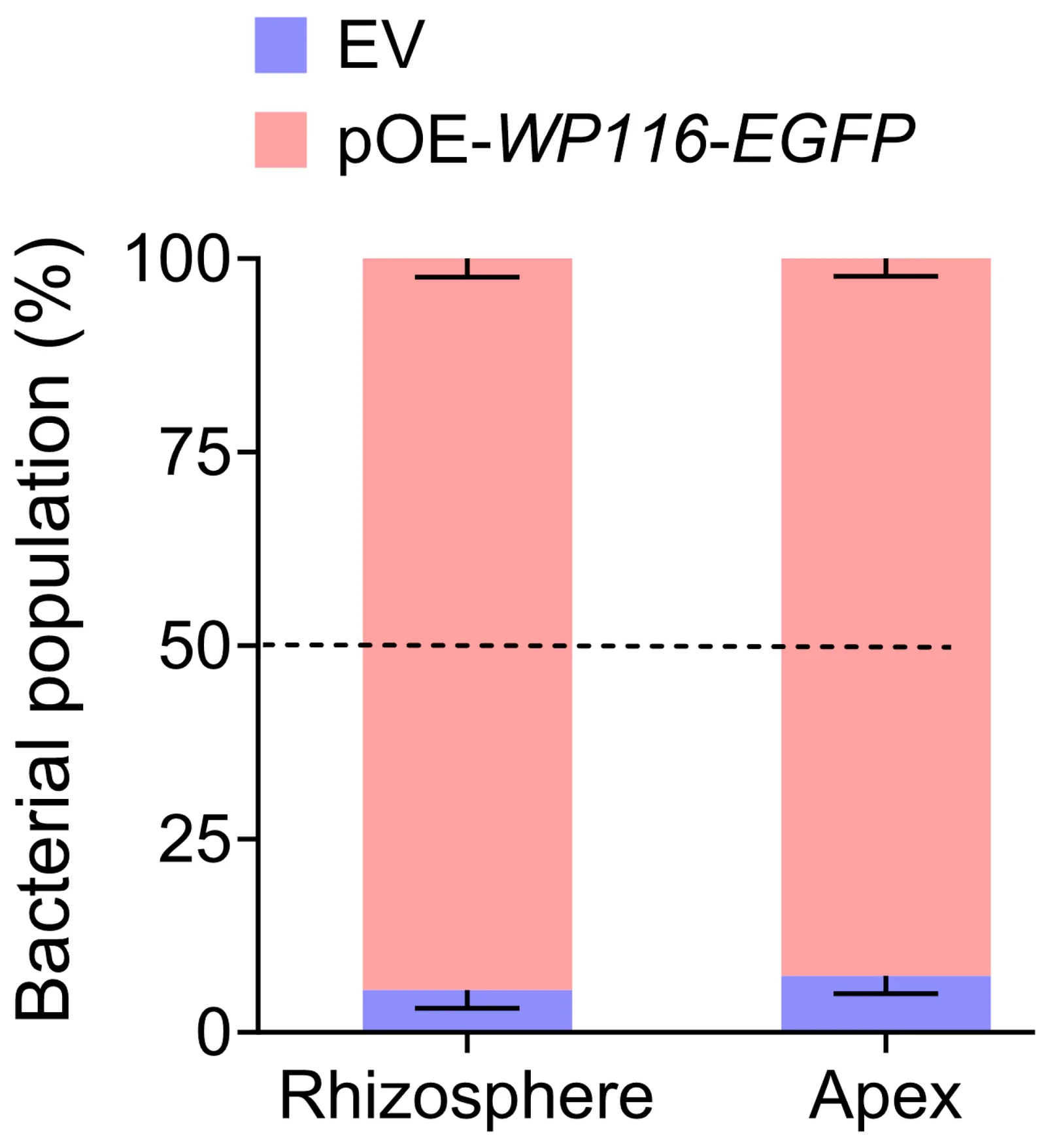
Competitive root colonization of the *WP116*-overexpression and EV strains derived from *Pseudomonas* sp. UW4. Bacteria were recovered from either the rhizosphere or root tips of wheat plants 10 days after co-inoculation of equal amounts of the *WP116*-overexpression strain and the EV strain into the wheat rhizosphere. EV, empty-vector control strain; pOE-*WP116*-*EGFP*, *WP116*-overexpression strain. Data are presented as means ± SD (n = 8 plants). In both the rhizosphere and root apex, differences between the *WP116*-overexpression strain and the EV strain were significant according to Student’s *t*-test on paired samples (*p* < 0.05).

Equal amounts of the *WP116*-overexpression strain and the EV strain were inoculated into the wheat rhizosphere for competitive rhizosphere colonization assays, and the relative proportion of each strain in the total population was assessed after 10 days. In both the rhizosphere and root tips, the *WP116*-overexpression strain predominated, accounting for more than 90% of the bacterial population and exhibiting a markedly 9-fold higher abundance than the EV strain (Figure 7).

## 4. Discussion

This study provides the first report that ACC, a metabolism-dependent chemoattractant of *Pseudomonas* sp. UW4, induced the up-regulation of several chemoreceptor genes, including the ACC-specific receptor gene, which exhibited the highest increase at 1.4-fold (Figure 2). However, proteomic analysis revealed no significant changes in the protein abundance of any chemoreceptors in UW4. Instead, differentially expressed proteins were primarily enriched in pathways related to metabolism, growth, and motility (Table 1). The discrepancy between transcript and protein levels suggests that the abundance of chemoreceptor proteins in bacterial cells may be strictly controlled to maintain stability and buffering capacity.

The fact that transcriptional up-regulation did not translate into a corresponding increase in protein abundance implies that chemoreceptors may be governed by multi-level regulatory mechanisms—including post-transcriptional regulation, translation efficiency, and protein degradation/turnover—to maintain intracellular stability [28,29]. This homeostatic maintenance is crucial for ensuring the precision and robustness of the chemotactic signaling system, preventing signaling imbalances that could result from drastic fluctuations in receptor abundance [30].

In stark contrast to the relatively stable abundance of chemotactic receptors, ACC treatment upregulated five major enzyme systems associated with carbon and ACC metabolism, the TCA cycle, oxidative metabolism and the degradation of aromatic amino acids and compounds (Table 1). These findings suggest that ACC treatment is associated with extensive metabolic reprogramming in UW4. Specifically, the upregulation of AcdS may facilitate the cleavage of ACC into α-ketobutyrate and ammonia, thereby potentially providing carbon and nitrogen sources for the cell [31,32]. It is consistent with our previous study, in which ACC treatment upregulated *acdS* expression and concomitantly increased AcdS activity [23]. The concomitant upregulation of transketolase, a key enzyme in the pentose phosphate pathway (PPP) [33], may contribute to the production of ribose and NADPH, thereby supporting biosynthetic and metabolic processes associated with ACC utilization. Collectively, these coordinated proteomic changes suggest that UW4 undergoes substantial metabolic remodeling in response to ACC, which may contribute to the efficient utilization of ACC-derived nutrients.

Furthermore, ACC triggered a pre-adaptive response of *Pseudomonas* sp. UW4 to the rhizosphere niche as a rhizosphere signaling molecule. The following evidence supports this inference: First, the upregulation of enzyme systems involved in degrading aromatic amino acids and phenolic compounds suggests that the bacterium is preparing to utilize the abundant aromatic compounds in plant root exudates [34,35]. Second, the increased expression of proteins associated with colonization, adhesion and biofilm formation may indicate that the bacterium has initiated programs for surface colonization and attachment [36,37]. Such extensive transcriptional and translational reprogramming is likely to cause a substantial accumulation of nascent polypeptides within the cell, thereby elevating the risk of protein misfolding and oxidative stress. Accordingly, the coordinated upregulation of proteins involved in acute protein folding and oxidative stress responses is of clear physiological significance. To mitigate these stresses, the induction of peptidyl-prolyl isomerases (PPIases) may facilitate the efficient folding of nascent polypeptides [38], thioredoxin family proteins may help maintain intracellular redox homeostasis [39], and the TolC efflux pump may facilitate the export of toxic by-products generated during enhanced metabolic activity [40].

In contrast to the aforementioned upregulation, ACC downregulated proteins associated with flagellar assembly and motility, cell wall synthesis and cell division, and cell membrane and surface antigen synthesis. This phenomenon may reflect UW4’s active strategy to ‘shed’ flagella, cease swimming, decelerate cell proliferation and pause the renewal of the outer membrane and surface antigens upon sensing rhizosphere signals. This may conserve energy by reallocating limited metabolic resources toward metabolic reprogramming and preparation for colonization. On the other hand, it may facilitate evasion of plant immune recognition by altering surface antigenic determinants [41,42]. This transition in physiology from a ‘growth/motility-first’ to an ‘adaptation/colonization-first’ mode likely reflects a sophisticated bacterial strategy that resource reallocation and optimizes energy trade-off [43,44]. This rapid response mode, characterized by ‘sacrificing short-term expansion for metabolic reprogramming’, may enable UW4 to optimize resource utilization and establish a metabolic state conducive to subsequent colonization. Ultimately, this results in a significant long-term increase in the overall growth rate and biomass, which is consistent with our experimental observations of a 21.1% increase in maximum specific growth rate and a 3.6% increase in maximum biomass (Figure 1).

Notably, although ACC induced the transcriptional upregulation of certain chemoreceptor genes in *Pseudomonas* sp. UW4, it did not alter the abundance of receptor proteins. In contrast, this study successfully overcame this homeostatic constraint by trans-overexpressing *WP116* using a plasmid. WP116 receptor protein abundance increased 32.3-fold compared to the empty vector control strain, resulting in a 2-fold increase in total receptor abundance. The proportion of WP116 receptor in the total receptor pool increased from 1.7% to 27.3% (Figure 5), with all overexpressed receptors correctly localizing to the cell poles (Figure 4). Consequently, the chemotactic response of the *WP116*-overexpressing strain to ACC was enhanced: at a low concentration of 0.10 μM, its chemotaxis intensity was five times higher than that of the empty-vector control strain (Figure 6). Furthermore, the *WP116*-overexpression strain displayed enhanced competitiveness for wheat rhizosphere colonization. Upon co-inoculation with the empty-vector strain, it accounted for more than 90% of the bacterial population in both the rhizosphere and root tips, showing a more than 9-fold higher abundance than the EV strain (Figure 7).

To determine whether overexpression of the ACC chemoreceptor introduced physiological artifacts, we first compared the growth curves of the wild-type strain UW4, the WP116-overexpressing strain (pOE-*WP116*-*EGFP*), and the empty-vector control strain in LB medium. No significant differences in growth were observed among the three strains (Figure S1), indicating that plasmid carriage did not impose a detectable metabolic burden on the cells. We then performed chemotaxis drop assays to evaluate their responses to ACC. The diameters of the ACC chemotactic rings were indistinguishable between the wild-type UW4 and the empty-vector control strain, whereas both were smaller than that of the *WP116*-overexpressing strain (Figure S2). These results suggest that the enhanced ACC chemotactic response of the overexpression strain is primarily attributable to elevated WP116 expression rather than nonspecific effects associated with plasmid carriage or physiological perturbation.

The underlying mechanism for the overexpressing ACC chemoreceptor to enhance the chemotactic rhizocompetence of UW4 may be that the continuous ACC efflux secreted by roots provides AcdS-producing PGPR with a preferred chemoattractant and nutrient source [45]; consequently, the ACC chemoreceptor overexpressing strain, with its enhanced chemotactic response to ACC, demonstrated superior chemotactic rhizocompetence. These results demonstrate that cell poles have significant spatial capacity for anchoring transmembrane receptors and that increasing receptor abundance directly enhances a strain’s responsiveness to the corresponding chemoattractant. These findings suggest that receptor homeostasis, as evolved in nature, may serve as a survival strategy. However, disrupting this balance through artificial intervention allows for the targeted enhancement of specific PGPR functions, providing a vital technological avenue for rhizosphere microbiome engineering.

## 5. Conclusions

In summary, quantitative analyses at transcriptional and proteomic levels demonstrate that, for the model PGPR strain *Pseudomonas* sp. UW4, ACC serves as a metabolic substrate and chemoattractant, but also as a key signaling molecule that triggers the transition to a lifestyle suited to colonizing the rhizosphere. This transition is manifested at multiple levels: at the transcriptional level, ACC selectively induces the expression of chemoreceptor genes while maintaining stable receptor protein levels via homeostatic mechanisms; at the proteomic level, ACC reprograms carbon, nitrogen and energy metabolism; and at the physiological level, ACC promotes a shift in bacteria from a motile state to a colonizing state. Furthermore, trans-overexpression of the ACC chemoreceptor disrupts this homeostatic balance by elevating receptor abundance. This consequently enhances UW4’s chemotactic response to ACC and its ability to competitively colonize the rhizosphere.

These findings deepen our understanding of the molecular mechanisms underlying metabolism-dependent chemotaxis [46,47] and provide a theoretical foundation for applying synthetic biology strategies to enhance the rhizosphere colonization capability and plant growth-promoting efficacy of PGPR. Furthermore, the rational utilization of ACC as a chemotactic signal, combined with the engineering of PGPR strains with enhanced ACC chemotactic responses, represents a promising strategy to improve their competitive colonization in the plant rhizosphere. This approach may facilitate the development of more efficient PGPR-based technologies for promoting plant growth and contributing to sustainable agriculture.

## Figures and Tables

**Table 1:** 1-Aminocyclopropane-1-carboxylate-induced differential protein expression in *Pseudomonas* sp. UW4.

NCBI Accession No.	Description ^a^	Fold Change
WP_015096486	Transketolase	21.15
WP_015096487	Acc deaminase	7.39
WP_015096483	Dihydrolipoyllysine-residue acetyltransferase	4.92
WP_015093534	Homogentisate 1,2-dioxygenase	4.82
WP_015093535	Fumarylacetoacetase	3.90
WP_007979060	4-hydroxyphenylpyruvate dioxygenase	3.18
WP_015093536	Maleylacetoacetate isomerase	3.09
WP_015096484	Alpha-ketoglutarate dehydrogenase	2.86
WP_015097108	3′-5′ exonuclease	2.76
WP_015094038	Duf1127 domain-containing protein	2.27
WP_015092956	Slipin family protein	2.10
WP_007975646	Ybak/ebsc family protein	2.09
WP_015094713	Hypothetical protein boo89_01065	2.05
WP_015095354	Duf5629 family protein	2.04
WP_015096023	Xanthine dehydrogenase accessory protein xdhc	1.94
WP_015096417	Flp pilus assembly protein cpab	1.90
WP_049810835	Hypothetical protein boo89_04305	1.87
WP_015094545	Nad-dependent epimerase	1.87
WP_007975977	Hypothetical protein	1.78
WP_015096319	Ketosteroid isomerase-like protein	1.77
WP_015096315	Tolc family outer membrane protein	1.69
WP_015092895	Surf1 family protein	1.67
WP_144443095	Formate dehydrogenase-n subunit alpha	1.66
WP_015095051	Duf1652 domain-containing protein	1.65
WP_015096334	Thioredoxin family protein	1.65
WP_015095088	Peptidylprolyl isomerase	1.62
WP_015095127	Amp-binding protein	1.61
WP_015095128	(2Fe-2S)-binding protein	1.60
WP_015093732	Pilin assembly protein	1.59
WP_015094782	2-oxoglutarate and iron-dependent oxygenase domain-containing protein	1.59
WP_015095303	Duf3203 family protein	1.58
WP_007972517	Agmatinase	1.55
WP_015096314	Retention module-containing protein	1.54
WP_015094489	Duf2388 domain-containing protein	1.52
WP_015094348	Duf3077 domain-containing protein	0.66
WP_015096764	Putative lipid ii flippase ftsw	0.64
WP_015096893	Murein biosynthesis integral membrane protein murj	0.60
WP_015095974	O-antigen ligase family protein	0.53
WP_041474761	Fusaric acid resistance protein	0.47
WP_010207715	Membrane protein insertion efficiency factor YidD	0.45
WP_015096279	Flagellar assembly peptidoglycan hydrolase FlgJ	0.003

^a^ Protein descriptions were accessed from the UniProt database (website: https://www.uniprot.org/, accessed on 12 May 2026) and NCBI database (website: https://www.ncbi.nlm.nih.gov/, accessed on 12 May 2026).

## Data Availability

Data are contained within the article and Supplementary Materials.

## Supplementary Materials

The following supporting information can be downloaded at https://www.mdpi.com/article/10.3390/microorganisms14081632/s1, Table S1: RT-qPCR primers for 15 genes encoding extracellular LBD-containing chemoreceptors and the *gyrB* reference gene; Table S2: Sequences of PCR primers used in this study; Table S3: Proteomic profiles of *Pseudomonas* sp. UW4 in LB + ACC and LB; Table S4: Proteomic profiles of *Pseudomonas* sp. UW4/pOE-*WP116*-*EGFP* and the empty-vector control strain; Figure S1. Validation of *gyrB* as the reference gene for RT-qPCR; Figure S2. Growth curves of three *Pseudomonas* sp. UW4 strains cultured in LB medium; Figure S3. Chemotactic responses of three *Pseudomonas* sp. UW4 strains toward ACC measured using the chemotaxis drop assay.

## References

[1] Colin R., Ni B., Laganenka L., Sourjik V. (2021). Multiple functions of flagellar motility and chemotaxis in bacterial physiology. FEMS Microbiol. Rev..

[2] Wadhams G.H., Armitage J.P. (2004). Making sense of it all: Bacterial chemotaxis. Nat. Rev. Mol. Cell Biol..

[3] Hazelbauer G.L., Falke J.J., Parkinson J.S. (2008). Bacterial chemoreceptors: High-performance signaling in networked arrays. Trends Biochem. Sci..

[4] Briegel A., Ortega D.R., Tocheva E.I., Wuichet K., Li Z., Chen S., Müller A., Iancu C.V., Murphy G.E., Dobro M.J. (2009). Universal architecture of bacterial chemoreceptor arrays. Proc. Natl. Acad. Sci. USA.

[5] Hazelbauer G.L., Engstrom P. (1981). Multiple forms of methyl-accepting chemotaxis proteins distinguished by a factor in addition to multiple methylation. J. Bacteriol..

[6] Hazelbauer G.L., Engstrom P., Harayama S. (1981). Methyl-accepting chemotaxis protein III and transducer gene Trg. J. Bacteriol..

[7] Zatakia H.M., Arapov T.D., Meier V.M., Scharf B.E. (2018). Cellular stoichiometry of methyl-accepting chemotaxis proteins in *Sinorhizobium meliloti*. J. Bacteriol..

[8] Cannistraro V.J., Glekas G.D., Rao C.V., Ordal G.W. (2011). Cellular stoichiometry of the chemotaxis proteins in *Bacillus subtilis*. J. Bacteriol..

[9] López-Farfán D., Reyes-Darias J.A., Krell T. (2017). The expression of many chemoreceptor genes depends on the cognate chemoeffector as well as on the growth medium and phase. Curr. Genet..

[10] López-Farfán D., Reyes-Darias J.A., Matilla M.A., Krell T. (2019). Concentration dependent effect of plant root exudates on the chemosensory systems of *Pseudomonas putida* KT2440. Front. Microbiol..

[11] Matilla M.A., Genova R., Martín-Mora D., Maaβ S., Becher D., Krell T. (2023). The cellular abundance of chemoreceptors, chemosensory signaling proteins, sensor histidine kinases, and solute binding proteins of *Pseudomonas aeruginosa* provides insight into sensory preferences and signaling mechanisms. Int. J. Mol. Sci..

[12] Matilla M.A., Velando F., Tajuelo A., Martín-Mora D., Xu W., Sourjik V., Gavira J.A., Krell T. (2022). Chemotaxis of the human pathogen *Pseudomonas aeruginosa* to the neurotransmitter acetylcholine. mBio.

[13] Hida A., Oku S., Miura M., Matsuda H., Tajima T., Kato J. (2020). Characterization of methyl-accepting chemotaxis proteins (MCPs) for amino acids in plant-growth-promoting rhizobacterium *Pseudomonas protegens* CHA0 and enhancement of amino acid chemotaxis by mcp genes overexpression. Biosci. Biotechnol. Biochem..

[14] Rico-Jiménez M., Roca A., Krell T., Matilla M.A. (2022). A bacterial chemoreceptor that mediates chemotaxis to two different plant hormones. Environ. Microbiol..

[15] Boyeldieu A., Ali Chaouche A., Méjean V., Jourlin-Castelli C. (2021). Combining two optimized and affordable methods to assign chemoreceptors to a specific signal. Anal. Biochem..

[16] Park J., Aminzare Z. (2021). A mathematical description of bacterial chemotaxis in response to two stimuli. Bull. Math. Biol..

[17] Kalinin Y., Neumann S., Sourjik V., Wu M. (2010). Responses of *Escherichia coli* bacteria to two opposing chemoattractant gradients depend on the chemoreceptor ratio. J. Bacteriol..

[18] Li J., Ovakim D.H., Charles T.C., Glick B.R. (2000). An ACC deaminase minus mutant of *Enterobacter cloacae* UW4 no longer promotes root elongation. Curr. Microbiol..

[19] Glick B.R., Karaturovíc D.M., Newell P.C. (1995). A novel procedure for rapid isolation of plant growth promoting pseudomonads. Can. J. Microbiol..

[20] Duan J., Jiang W., Cheng Z., Heikkila J.J., Glick B.R. (2013). The complete genome sequence of the plant growth-promoting bacterium *Pseudomonas* sp. UW4. PLoS ONE.

[21] Chai R., Li F., Gao Y., Liu D., Shang D., Yang Y., Yu J., Zhou C., Li Y., Song A. (2024). Unveiling preferred chemoattractants for rhizosphere PGPR colonization by molecular docking and molecular dynamics simulations. Comput. Electron. Agric..

[22] Li Y., Li Y., Wang Y., Yang Y., Qi M., Su T., Li R., Liu D., Gao Y., Qi Y. (2024). Flg22-facilitated PGPR colonization in root tips and control of root rot. Mol. Plant Pathol..

[23] Gao X., Li T., Liu W., Zhang Y., Shang D., Gao Y., Qi Y., Qiu L. (2020). Enhancing the 1-aminocyclopropane-1-carboxylate metabolic rate of *Pseudomonas* sp. UW4 intensifies chemotactic rhizocompetence. Microorganisms.

[24] Skinner S.O., Sepúlveda L.A., Xu H., Golding I. (2013). Measuring mRNA copy number in individual *Escherichia coli* cells using single-molecule fluorescent in situ hybridization. Nat. Protoc..

[25] Darias J.A.R., García-Fontana C., Lugo A.C., Rico-Jiménez M., Krell T., Filloux A., Ramos J.L. (2014). Qualitative and quantitative assays for flagellum-mediated chemotaxis. Pseudomonas Methods and Protocols.

[26] Liu X., Wood P.L., Parales J.V., Parales R.E. (2009). Chemotaxis to pyrimidines and identification of a cytosine chemoreceptor in *Pseudomonas putida*. J. Bacteriol..

[27] Matilla M.A., Espinosa-Urgel M., Rodríguez-Herva J.J., Ramos J.L., Ramos-González M.I. (2007). Genomic analysis reveals the major driving forces of bacterial life in the rhizosphere. Genome Biol..

[28] Cardozo M.J., Massazza D.A., Studdert C.A., Parkinson J.S. (2010). Disruption of chemoreceptor signalling arrays by high levels of CheW, the receptor–kinase coupling protein. Mol. Microbiol..

[29] Parkinson J.S., Hazelbauer G.L., Falke J.J. (2015). Signaling and sensory adaptation in *Escherichia coli* chemoreceptors: 2015 update. Trends Microbiol..

[30] Sourjik V., Wingreen N.S. (2012). Responding to chemical gradients: Bacterial chemotaxis. Curr. Opin. Cell Biol..

[31] Glick B.R. (2014). Bacteria with acc deaminase can promote plant growth and help to feed the world. Microbiol. Res..

[32] Glick B.R., Cheng Z., Czarny J., Duan J. (2007). Promotion of plant growth by ACC deaminase-producing soil bacteria. Eur. J. Plant Pathol..

[33] Stincone A., Prigione A., Cramer T., Wamelink M.M., Campbell K., Cheung E., Olin-Sandoval V., Grüning N.-M., Krüger A., Tauqeer Alam M. (2015). The return of metabolism: Biochemistry and physiology of the pentose phosphate pathway. Biol. Rev..

[34] Bais H.P., Weir T.L., Perry L.G., Gilroy S., Vivanco J.M. (2006). The role of root exudates in rhizosphere interactions with plants and other organisms. Annu. Rev. Plant Biol..

[35] Sasse J., Martinoia E., Northen T. (2018). Feed your friends: Do plant exudates shape the root microbiome?. Trends Plant Sci..

[36] O’Toole G.A., Kolter R. (1998). Flagellar and twitching motility are necessary for *Pseudomonas aeruginosa* biofilm development. Mol. Microbiol..

[37] Tomich M., Planet P.J., Figurski D.H. (2007). The *tad* locus: Postcards from the widespread colonization island. Nat. Rev. Microbiol..

[38] Schiene-Fischer C. (2015). Multidomain peptidyl prolyl cis/trans isomerases. Biochim. Biophys. Acta. Gen. Subj..

[39] Lu J., Holmgren A. (2014). The thioredoxin antioxidant system. Free Radic. Biol. Med..

[40] Zgurskaya H.I., Krishnamoorthy G., Ntreh A., Lu S. (2011). Mechanism and function of the outer membrane channel TolC in multidrug resistance and physiology of enterobacteria. Front. Microbiol..

[41] Boller T., Felix G. (2009). A renaissance of elicitors: Perception of microbe-associated molecular patterns and danger signals by pattern-recognition receptors. Annu. Rev. Plant Biol..

[42] Newman M.A., Sundelin T., Nielsen J.T., Erbs G. (2013). MAMP (microbe-associated molecular pattern) triggered immunity in plants. Front. Plant Sci..

[43] Basan M., Hui S., Okano H., Zhang Z., Shen Y., Williamson J.R., Hwa T. (2015). Overflow metabolism in *Escherichia coli* results from efficient proteome allocation. Nature.

[44] Scott M., Gunderson C.W., Mateescu E.M., Zhang Z., Hwa T. (2010). Interdependence of cell growth and gene expression: Origins and consequences. Science.

[45] Li T., Zhang J., Shen C., Li H., Qiu L. (2019). 1-Aminocyclopropane-1-carboxylate: A novel and strong chemoattractant for the plant beneficial rhizobacterium *Pseudomonas putida* UW4. Mol. Plant-Microbe Interact..

[46] Lacal J., García-Fontana C., Muñoz-Martínez F., Ramos J.L., Krell T. (2010). Sensing of environmental signals: Classification of chemoreceptors according to the size of their ligand binding regions. Environ. Microbiol..

[47] Sampedro I., Parales R.E., Krell T., Hill J.E. (2015). *Pseudomonas* chemotaxis. FEMS Microbiol. Rev..

